# Neurodegeneration and acute neuronal injury drastically skew the p‐tau217/non‐p217 ratio in both CSF and plasma regardless of analytical technique – a mass spectrometry and Simoa study

**DOI:** 10.1002/alz.094606

**Published:** 2025-01-09

**Authors:** Kaj Blennow

**Affiliations:** ^1^ Department of Psychiatry and Neurochemistry, Institute of Neuroscience and Physiology, The Sahlgrenska Academy, University of Gothenburg, Mölndal, Sweden; Department of Psychiatry and Neurochemistry, Institute of Neuroscience and Physiology, The Sahlgrenska Academy at the University of Gothenburg, Mölndal Sweden

## Abstract

**Background:**

Plasma p‐tau217 shows high promise as an AD biomarker. In some mass spectrometry (MS) studies, the ratio between p‐tau217 and the corresponding non‐phospho peptide tau212‐221 (p‐tau217/T217) was suggested to increase accuracy to detect AD‐type tau phosphorylation. However, disorders with rapid neurodegeneration or acute neuronal injury, are known to have increased neurodegeneration biomarkers e.g., total tau [T‐tau] and brain‐derived tau [BD‐tau]. Since both T‐tau and BD‐tau contain the T217 peptide sequence, it is likely that T217 also would increase, and the ratio thus be affected, but CSF/plasma p‐tau217/T217 has not been studied in these conditions.

**Method:**

CSF and plasma p‐tau217, T217 and p‐tau217/T217 measured by Simoa and/or MS; T‐tau and BD‐tau by Simoa. Neurodegeneration cohorts (n>300) included CSF and plasma from controls, AD and sporadic Creutzfeldt‐Jakob disease (CJD). Plasma samples from acute neuronal injury cohorts (n>500) included traumatic brain injury (TBI) and cardiac arrest with brain hypoxia (Simoa analyses) plus ischemic stroke (MS and Simoa).

**Result:**

Both p‐tau217 and the p‐tau217/T217 ratio markedly increased in AD, in CSF around 400% and in plasma around 280% of mean control levels. All neurodegeneration biomarkers increased dramatically in CJD (e.g. 1400% for CSF T217 by MS) and with acute brain injury (e.g. 600% increase in TBI for plasma BD‐tau). Changes were similar when measured by MS or Simoa, and CSF T217 by MS also correlated tightly with T‐tau by Lumipulse (r = 0.99). Consequently, in both CSF and plasma, the p‐tau217/T217 ratio was drastically skewed in rapidly progressive dementias (e.g., 16% of control levels in CSF in CJD) and in acute brain conditions, with marked reductions in p‐tau217 ratios in CSF and plasma, regardless of analytical technique.

**Conclusion:**

Non‐phosphorylated tau variants (T‐tau, BD‐tau and T217) all markedly increase in dementias with rapid neurodegeneration and with acute brain injury and thereby drastically skew the p‐tau217/T217 ratio. This will be an important consideration for applications in clinical routine where patients with mixed pathologies are common. Therefore, plasma p‐tau217 alone may be preferred, as also supported by recent studies (Montoliu‐Gaya, Acta Neuropathol 2024;147:5) showing similar performance to identify AD for plasma p‐tau217/T217 and p‐tau217 by itself.

